# A framework for participatory work environment interventions in home care – success factors and some challenges

**DOI:** 10.1186/s12913-022-07710-2

**Published:** 2022-03-15

**Authors:** Johanna Persson, Gerd Johansson, Inger Arvidsson, Britt Östlund, Charlotte Holgersson, Roger Persson, Christofer Rydenfält

**Affiliations:** 1 Department of Design Sciences, Lund University, Lund, Sweden; 2 Division of Occupational and Environmental Medicine, Lund University, Lund, Sweden; 3 Department of Biomedical Engineering and Health Systems, KTH Royal Institute of Technology, Stockholm, Sweden; 4 Department of Industrial Economics and Management, KTH Royal Institute of Technology, Stockholm, Sweden; 5 Department of Psychology, Lund University, Lund, Sweden

**Keywords:** Home care, Work environment, Participation, Intervention, Action research, Action learning, Systematic evaluation

## Abstract

**Background:**

Home care is beset with work environment issues and high staff turnover, while research concerned with interventions to improve the work environment is sparse. Few of the existing interventions apply a participative approach, despite this being associated with more positive outcomes and sustainable change. This paper presents a framework, rooted in action research and action learning, for participatory work environment interventions in home care, and demonstrates how this framework has been implemented in four Swedish home care organizations.

**Methods:**

The framework has three phases (pre-intervention, intervention planning and intervention implementation) and consists of cycles of action and reflection in three constellations: a *group of researchers*, a *reference group* with labour market organization representatives and home care managers, and *intervention work groups* in the home care organizations. The work was documented and analysed with focus on the realization of the framework and challenges that were met on the way. The interventions were evaluated using a pre-/post-test questionnaire design.

**Results:**

Parts of the framework were successfully implemented. The pre-intervention phase and the intervention planning phase, with intervention work groups, worked well. All four groups identified one intervention relevant to their own context. However, only two of the proposed interventions were fully implemented and evaluated. The high staff and management turnover, and the high rate of organizational changes made it impossible to evaluate the interventions statistically. Yet, data from open-ended questions in the post questionnaire showed that the two implemented interventions were perceived as successful.

**Conclusions:**

The participatory framework, presented in this paper, seems promising for work environment interventions in home care. The framework was designed to reduce the risk of known disturbances affecting the process in unstable organizations. Despite this, it proved challenging to execute the framework, and especially the interventions, due to changes happening at high speed. In the two cases where organizational changes were not dominating, the interventions were implemented successfully. While the prerequisites for participation and successful implementation could be improved somewhat, the main issue, the instability of the organizational context, is hard for researchers or the individual home care units to tackle alone.

## Background

The older population is growing, and although many older persons live a healthy and active life, there is also a changed disease pattern with a higher degree of co-morbid illness and disease, polypharmacy and increased needs for long-term care [1]. Accordingly, more care and more services are delivered in people’s homes. To meet these changing needs, the workforce in the home care sector needs to increase, which is a challenge for a sector characterized by several work environment–related problems, recruitment difficulties and high staff turnover [2–10].

The present study targets assistant nurses and home care aides employed by municipalities to provide home help services and basic care in the residents’ own homes.^1^ Historically, this type of work has been unpaid, mainly performed by women caring for family members, but it is now publicly financed in many countries [11–13]. In Sweden, which is the setting for this study, home care assistant nurses and aides constitute one of the largest groups in the working population, and 92% of them are women [14].

Despite the above-mentioned work environmental issues, the research concerned with interventions intended to improve the work environment in home care is sparse. A recent review by Rydenfält et al. [15] points out that the studies that do exist tend to focus on minor adjustments, such as changing a specific behaviour or introducing new technology, ignoring more system-wide and urgent issues such as stress, sick leave or the low status of the sector. Additionally, it shows that employee participation is a mechanism associated with successful interventions in home care.

Participation is related to the concept of empowerment [16–19], and previous studies show that the feeling of empowerment at work is associated with an increased job satisfaction [20–22]. Participation in the development of interventions is considered to lead to a better fit between the intervention and the problems experienced by the employees [23], and it is furthermore associated with a decreased resistance to change [24]. In organizations associated with high demands, low control, low social support and isolation, which home care exemplifies, aspects related to the feeling of work engagement and sense of coherence are considered especially important to achieve positive organizational change [3, 25–27].

The benefits of participation and staff engagement are thus twofold. On the one hand, it could help ensure the relevance of the intervention, and on the other hand, it could, due to empowerment, have a positive effect on job satisfaction. For home care, there is a shortage of research in this area, and there are few available studies that adopt a participatory approach to organizational improvement [28, 29]. For these reasons, given the inattention to the work environment in the home care sector, there is a need for more research concerned with participation in work environment–related organizational changes in the home care sector.

In this article, we present an action-oriented framework for participation intended to facilitate sustainable work environment changes in home care practice. In the coming sections, the proposed framework is introduced, followed by a description of its actual implementation in four home care organizations. Finally, we discuss outcomes and challenges associated with the application of participation in the home care setting.

### A framework for participatory work environment interventions

The participatory intervention framework presented here is rooted in the action-oriented tradition, that is, in action research and action learning [30–32]. Specifically, the framework is influenced by action learning in the sense that it promotes a cyclic process of action and reflection in seeking pragmatic and meaningful solutions to identified issues and increasing knowledge on how to approach similar situations further on [33]. In an organization, this typically implies that a *set* of *people* come together to address problems from their own context and approach them in a reflective manner to develop both the people and the organization [34]. Another aspect of the framework is that it puts emphasis on experiential learning, since it is the participants’ own experiences that form the starting point for learning, and new knowledge is created through reflection on experience and on the systematic testing and collection of new experiences [35].

However, the current framework *goes beyond* action learning in at least two ways; First, it includes inquiry into the wider problem domain from both a research and practice perspective, before the actual problems to be worked with and learned about are defined. This puts the work of the local set of people into a larger context, which in turn can lead to a wider applicability of the problems and solutions being defined. It also increases the likelihood of the solutions being novel, that is, decreases the likelihood of reinventing the wheel. Second, while the practitioners are the ones responsible for problem definition, the reflection on possible solutions and the actual actions intended to test those solutions, the framework provides an entirely research-based evaluation strategy for the solutions suggested. This is important, since there is a lack of evaluated work environment–related interventions in home care that could contribute to wider policy and organizational changes.

**Fig. 1:**
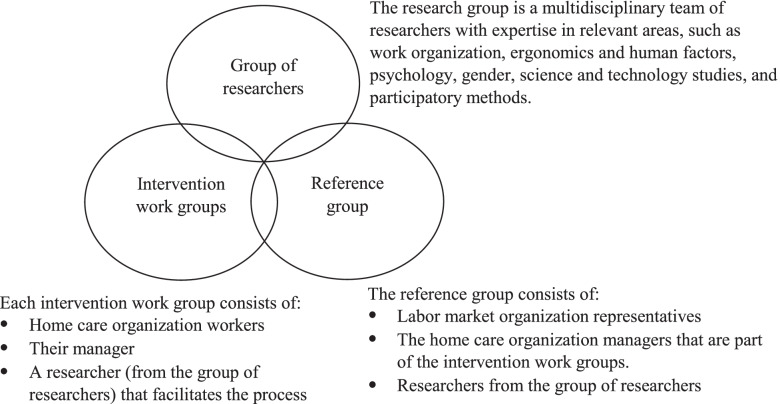
Overview of the different groups of actors in the framework, and their relationships

The framework consists of cycles of action and reflection in three constellations that represent different functions in the process: a *group of researchers*, a *reference group* with representatives from relevant stakeholders, such as labour market organizations and home care managers, as well as *intervention work groups* from home care organizations, with the latter being the set of people who frame problems that are locally relevant as well as plan and implement the interventions. There is an overlap between the three constellations in the sense that some of the researchers from the research group are members of the reference group. In the reference group there are also managers from the home care organizations, that are part of the intervention work groups. Researchers from the research group are furthermore acting as facilitators in the intervention work groups. An overview of these actors and their relationships is presented in Fig. 1. Each constellation goes through its own experiential learning cycle [35]. However, on certain points in the process, output from one constellation is handed over to another. This output is then *experienced* by the receiving constellation and becomes input to that constellation’s experiential learning.

Thus, the aims of the framework are to ensure (1) local relevance and ownership of the interventions developed, (2) that the interventions are based on prior knowledge about the problem domain, (3) that problems experienced on a sector/system level are taken into consideration when designing interventions and (4) that the interventions are properly evaluated, to be able to learn from and spread the results to others.

#### Three phases

**Fig. 2:**
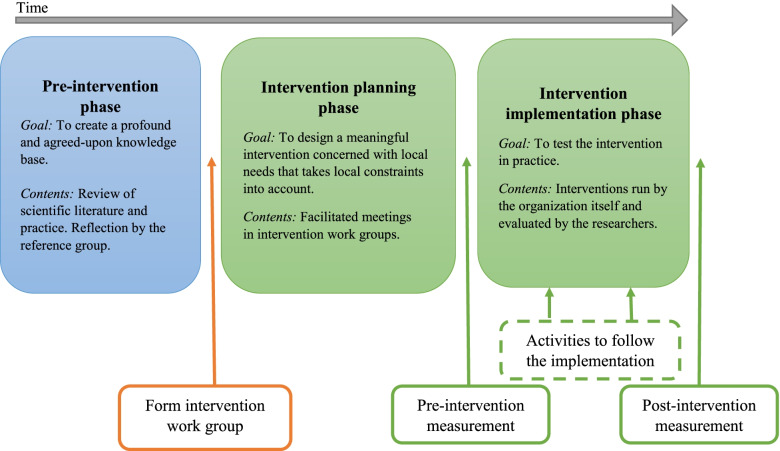
Overview of the three phases of the framework for participatory work environment interventions in home care

The framework has three phases: the *pre-intervention phase*, the *intervention planning phase* and the *intervention implementation phase,* as shown in Fig. 2.

The *pre-intervention phase* contains actions to build a solid knowledge base and identify present challenges and needs in home care on a societal level. It consists of data elicitation from both scientific literature and home care practice. It also consists of several iterations of reflection in the *reference group*, partly based on data assembled and analysed by the *research group* and partly based on the *reference group* members’ own experiences. In parallel, and prior to the *reference group*, the *research group* also goes through phases of reflection on the data being assembled and the outcomes from the *reference group*. The purpose of this phase is to ensure that the process benefits from knowledge and understanding gained by what others do or have done, as well as to ensure commitment through participation by the other members of the reference group, including labour market representatives and home care managers.

**Fig. 3:**
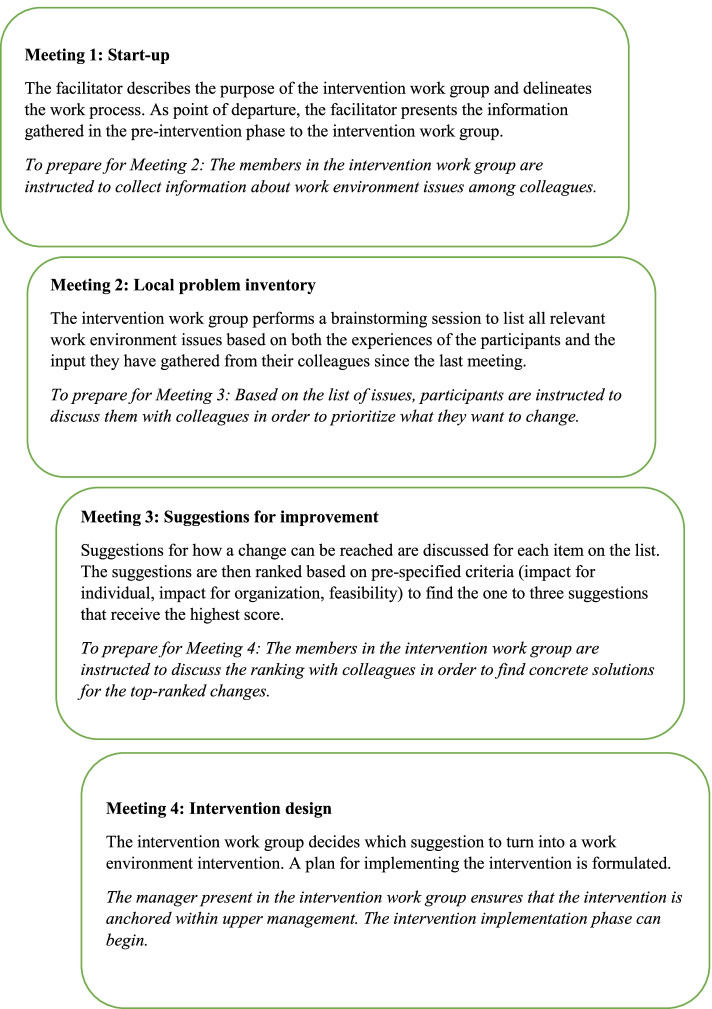
The scheme of the four facilitated intervention work group meetings in the intervention planning phase

In the *intervention planning phase*, intervention work groups are formed, with the goal of designing interventions for sustainable work environment changes in home care practice. Each home care organization form groups of six to eight people working in the home care organization, including a first-line manager for that organization (i.e. the manager with direct responsibility for the home care workers). Each organization chooses which people to include in the work group. This can, for example, be an even distribution of people representing different teams, or be based on their personal willingness to engage. A researcher, from the group of researchers, acts as facilitator and leads the intervention group from the first to the last meeting, where an intervention idea and a plan for implementation is decided. The group meets four times, following the structure in Fig. 3.

During the first meeting, the facilitator presents results from the pre-intervention phase for the group to reflect upon. The intervention that the work group eventually chooses does not need to be drawn from the input from the pre-intervention phase but can be freely chosen by the participants of the work group. The input from the pre-intervention phase is merely aimed to widen the perspectives. Together with the group members’ own experiences this step constitutes the beginning of the intervention work group’s experiential learning cycle [35]. Further on in the process, the facilitator takes a less prominent role in the group. To ensure a broader commitment and extend the level of participation beyond the intervention work group, the participants of the group are asked to, between each meeting, involve the rest of the work force in the process and collect their input. This is a way to promote engagement among staff outside of the intervention work group and make sure that everyone has an opportunity to contribute. It is also a test, in the sense that, the intervention work group members test, on the rest of the staff, the validity of both the work environment issues they have identified and the solutions to those problems. Once the intervention has been decided upon, the implementation phase follows.

The last phase is the *intervention implementation phase*. To measure the work environment baseline, a pre-intervention measurement is conducted as a first step in this phase, before the interventions start. The intervention is thereafter implemented and run for a suitable length of time, depending on the chosen intervention. The intervention is evaluated towards the end of the intervention with the same measurement as was used before the intervention. Activities to follow the implementation might be added as well. Since there is a lack of evaluated interventions concerning the work environment in home care, and improvements initiated by the practitioners rarely are evaluated systematically, the evaluation part of this step is important for building a larger evidence base [15, 36]. For that reason, the evaluation as far as possible should apply established and standardized instruments in combination with more context-specific instruments.

#### Actions to reduce the risk of disturbances to the participative process

Interventions are vulnerable to disturbances, and home care is associated with high staff turnover and high workload with not much time to work with tasks other than the care-related activities [37]. Several measures in the framework presented are intended to reduce the risk of disturbances to the participative process. Specifically, it is designed to:

- *Provide structure*: The framework consists of a set of predefined steps. The output from previous steps is used as input to the following steps and phases. The reference group is led by researchers who document and structure the work in the group. The intervention work group is furthermore facilitated by an external person to help the work group to identify issues in a structured way. Finally, the intervention itself is supported by a structured pre- and post-intervention evaluation instrument.
- *Ensure commitment*: To cater for long-term change, it is important that the whole organization is committed to participating [38]. To reach commitment on a higher level, stakeholders, such as labour market organization representatives, and higher-level managers participate in the reference group in order to foster their commitment in relation to the overall problem description. Furthermore, first-line managers participate in the local intervention work groups to ensure that the planned interventions are anchored within management and can be implemented. Actions are furthermore defined to ensure commitment of staff beyond those who are part of the intervention work groups. Before the process using the framework starts, the commitment of the participating organizations must be established.

In the following sections, we will present a case study that illustrates how the framework for participation described above can be implemented and some difficulties associated with the use of participative methods in home care.

## Methods

The study was designed as a case study where the participatory framework was tested in four home care organizations in southern Sweden. This approach is appropriate for investigating the implementation of the framework in the holistic, real-world context and to gain a wide knowledge on not only *how* the framework works in practice but also *why* it works in the observed way [39].

### Research setting

Besides the case organizations, that is, the ones actually intended to implement interventions, a reference group was created, consisting of managers from the four participating home care organizations, five labour market organization representatives from employer organizations and trade unions, and two researchers. The research group consisted of seven researchers with a vast joint expertise in areas including work organization, ergonomics and human factors, psychology, gender and science and technology studies. The group members also had long experience of participative methods.

To increase the potential amount of information collected, the four cases were chosen to ensure variation in certain dimensions [40]. Case 1 and case 2 were home care organizations located in two smaller, rural towns. Case 3 was located in the outskirts of a small city, and case 4 was situated in the central parts of a large city. Four researchers participated as facilitators, one in each of the four intervention work groups responsible for the planning of interventions. An overview of the four cases is found in Table 1. In addition to the actions designed into the framework to reduce the risk of disturbances to the participative process and to facilitate commitment, the case organizations were offered economic reimbursement, consisting of an amount covering approx. 4–6 weeks of work hours, to be distributed as desired.

**Table 1:** Overview of the four case organizations participating in the study

	**Municipality**	**Home care organization**	**Intervention work group**
Case 1	Small, rural town with approx. 15,000 inhabitants	The home care organization consists of six geographically distributed units	The intervention work group consisted of seven people in total, including their first-line manager. There was one person from each of the six home care units, two women and four men
Case 2	Small, rural town with approx. 15,000 inhabitants	The home care organization is organized in three geographically distributed day units plus one night unit serving the whole municipality	The intervention work group consisted of eight people including their first-line manager. There were four people from the day units, one person from the night unit and two care coordinators (coordinating the care for all units). One person was a man and the rest were women
Case 3	Small city with approx. 50,000 inhabitants	The home care organization consists of seven geographically distributed units. One specific unit, with 50 employees, participated in the study	The intervention work group consisted of eight people from the day and evening shifts of the unit and their first-line manager. All of them were women
Case 4	Large city with approx. 350,000 inhabitants	The home care organization in the entire city employs several thousands of people and is organized in geographically distributed units. The participating unit included about 35 employees, scheduled for daytime, evening and weekend shifts	The intervention work group consisted of seven people, including their first-line manager. All of them were women

The study was performed according to ethical standards guidelines and was approved by the Swedish Ethical Review Authority (No. 2018/840). Information about the study and the interventions was communicated to staff in the participating home care organisations, and informed consent was obtained from all subjects participating in (or being affected by) interventions, control groups, intervention work groups, and pre- and post evaluations.

### The three phases of the framework

There are some degrees of freedom in the framework that allow for choosing how to perform specific parts of the implementation in practice, namely, how to construct a knowledge base in the pre-intervention phase or how to evaluate the interventions in a structured way. The methods chosen in each of the three phases for this case study are listed below.

The *pre-intervention phase* serves to build a solid knowledge base and identify present challenges and needs in home care. In the present study, this phase involved three concrete activities to reach the goal of generating this knowledge base: (i) a scientific literature review, (ii) a national survey of work environment interventions in Swedish home care and (iii) regular meetings of the reference group. Based on their insight into home care practice on both a local and a national level, the reference group discussed and reflected on work environment–related issues in home care.

The *intervention planning phase* started with the formation of intervention work groups according to the information presented in Table 1. A researcher was assigned as facilitator to each of the work groups, and meetings were planned according to the scheme in Fig. 3.

In the *intervention implementation phase* a pre- and post-test questionnaire was used to evaluate the interventions. The questionnaire consisted of a number of established and validated instruments, context-specific questions as well as intervention-specific questions [41–43]. The post-test questionnaire included a set of open-ended questions specifically addressing the intervention performed.

### Data collection and analysis

The implementation of the framework in the four municipalities was documented through *field notes* and *observations* from the intervention work group meetings, and *meeting notes* from the reference group meetings. The field notes contained information about the intervention work group process, including observations on, for example, which participants were active in the discussions and whether there were hindrances or breakthroughs in the discussions. These notes also included information about the topics of the discussions, such as which work environment problems were raised and how the group prioritized among them.

Two themes have been central in the analysis. The first one is the analysis of *the implementation of the framework* itself. The second one is which *work environment issues* that were discussed and prioritized in the intervention work groups. The second theme is of explicit interest since the overall aim is to create sustainable and positive work environment changes. To identify the work environment issues discussed in the intervention work groups, the field notes from these meetings were coded using QSR NVivo 12, resulting in an overview of the work environment issues that were brought up and prioritized in the four cases.

The process for analysing the first theme, the implementation of the action-oriented, participatory framework, was not as straightforward, since the framework involves three phases (pre-intervention phase, intervention planning phase and intervention implementation phase), and three sets of people (the group of researchers, the reference group and the intervention work group in each of the cases), interacting in cycles of reflection and action. The accumulated experiences of the research group, together with observations and notes that were collected throughout the whole process of the implementation of the participatory framework, were analysed in a bottom-up process, where the main author, through interaction with data as well as with the other researchers, identified recurring or significant concepts in the data, both across cases and within a case [44]. These concepts were then discussed in retrospective conversations among the researchers, to confirm and prioritize among them.

The analysis focused on the realization of the framework for participatory work environment interventions, that is, how the proposed process (Fig. 1) was implemented in practice, and challenges that were met on the way. Detailed results of the actual interventions, including pre- and post-test questionnaire results, are presented elsewhere.

## Results

The pre-intervention phase started in early 2017 and the intervention planning phase in autumn 2018, with the intervention implementation phase taking place between autumn 2019 and spring 2020.

### The pre-intervention phase: a unified view of challenges and needs

The pre-intervention phase served to make sure that the work was founded in up-to-date knowledge of the home care field through a scientific literature review and a national survey in home care practice, and by working with the reference group to elicit the perspectives of all members of this group. *The literature review* showed that home care work is a sparsely researched sector, especially when it comes to interventions intended to improve the work environment (*Reference deleted for anonymity*). It was noted in the review that the majority of the interventions concerned changing specific behaviours, such as a training intervention for the adoption of new practices for safe lifts, or introducing new technology, such as mobile health systems specialized for the home care situation, rather than tackling complex issues such as sick leave, stress or gender inequality.

If the literature review indicated a lack of interventions, *the national survey* instead provided a multitude of examples of ongoing activities in home care practice (*Reference deleted for anonymity*). The conclusion from this survey is that many diverse initiatives are initiated and carried out with the intention to improve the work environment in the Swedish home care sector. The aims of these initiatives covered a wide spectrum. However, the content of the initiatives mirrored societal trends, rather than the actual needs identified by existing research. Furthermore, change initiatives are seldom evaluated or made accessible to stakeholders outside the organization, so there is an inadequate learning from the activities performed.

Based on their own experience and input from the researchers, the reference group worked with the data from the literature review and the national survey to conduct a comprehensive analysis of the work environment–related problems facing the home care sector. All the organizations represented, whether employer organizations, home care managers or trade unions, agreed upon the need to improve the work environment in home care to decrease staff turnover and work-related illness, and increase the learning from current activities.

In summary, there was a unified view on the challenges in the sector and a need of participatory interventions in the home care organizations to improve the work environment. There was, furthermore, consensus on a demand for systematized evaluation to improve the potential for learning.

### Intervention planning phase: engaged work groups and a multitude of ideas

#### Forming and engaging the intervention work group

Once an intervention work group was formed there were no obstacles with engaging the participants in the process and guiding them through the steps in the intervention planning phase, from identifying problems to suggesting solutions and designing an intervention. The experience shared by all four intervention work group facilitators was that all group participants agreed upon the description of the work environment, coming from the pre-intervention phase. Everyone was positive towards the task of investing in improving it.

The process as described in the framework (Fig. 2) was performed according to plan, with approximately one meeting per month, in all four cases. A typical meeting lasted for approximately two hours and was planned beforehand in order to make sure the specified tasks of the meeting were covered. This means that in four meetings the intervention work groups went from being presented with the knowledge base, to generating a problem inventory, to suggesting changes and designing the intervention. During the process it was however obvious that the four meetings were not enough to reach all the way in the last step and an additional meeting was needed in most cases, to set the final details in how to design and implement the intervention.

#### Participation beyond the intervention work group

As described in the framework, the participants were supposed to share the information from the group meetings and make sure that the rest of the staff members participated in the discussions and the design of the intervention. This was something that was difficult to ensure, and it was not always clear whether any specific interaction had taken place with other members of the staff and in what way. Only one group explicitly discussed strategies for how to engage the larger staff group, by, for example, using the monthly staff meetings, but it is not clear to what extent this was done in the end. Hence, it is not known to what extent the views in the intervention work group represented the entire staff or mainly the views of the group participants.

#### Manager’s role

The managers were recurring participants in cases 1 and 2, while participating only in some meetings in the other two cases. In case 2, where the manager was an active partner, this was positive in the sense that she sometimes brought relevant topics to the table and gave an infusion to the discussion. On the other hand, her participation might have been inhibiting of the others, since the manager sometimes dismissed a discussion by saying that a solution had already been tested or was not possible due to lack of resources. It is also possible that certain issues were not raised at all due to the presence of the manager. In the end of the process, the participation of the manager was central to be able to anchor the intervention that was designed and to create a valid plan for implementation in the next phase.

#### Work environment issues and chosen interventions

A range of work environment issues was raised in the intervention work groups according to the coding of the topics in the field notes. The subject that was discussed the most, in all four groups throughout the process, concerned issues related to *planning and scheduling*. The lack of continuity among staff caused the schedule to constantly be redesigned and the ordinary routes to be disrupted. Temporary staff had to be called in on short notice. However, many of these substitute workers were new to the work and could only take on less complex care recipients, leaving a heavier burden for regular staff. Other aspects related to planning and scheduling were also mentioned: an increasing number of alarms from care recipients during daytime, time for transportation between care recipients that was either too short or not included in the planning at all, and that all scheduled time for doing administrative tasks or work with designated areas of responsibility disappeared in the constant rescheduling.

The participants in the intervention work groups also raised issues related to *work organization*, including the size of the home care teams and the fact that the organization was constantly being changed, whether it being new management, a new organization of the teams, or new digital systems to document the work. The latter was also emphasized as an issue of its own, wherein *digital systems* are beneficial in many ways but the multitude of systems increases the administrative burden. *The social climate* and staff members not treating each other, or the care recipients, well, was also mentioned in all four intervention work groups. The high staff turnover, both among assistant nurses and aides as well as among managers, was mentioned as one reason for not being able to create a sense of companionship, which could have a negative effect on the social climate. The group in case 2 considered this problem so dominant that it was brought up in all meetings and eventually also ended up being the issue that was chosen for the intervention.

Table 2 gives an overview of each of the four cases, including the most pressing issues that the intervention work group identified and the intervention they decided upon to improve their work environment.

**Table 2:** An overview of the identified issues and chosen intervention in each of the four cases

Case 1	***A “backup” resource for unforeseen events*** The most pressing need was related to the high occurrence of temporary staff needed to cover up for absence of permanent members of the staff and for unforeseen events. Temporary staff had low knowledge about the care recipients and could only tend to more simple tasks, always leaving the heavier and complex tasks to the permanent staff. To meet this need, the group decided upon an intervention that involved recruiting one extra person who would not be on a fixed schedule but serve as a backup in the organization to cover for someone being away, to respond to alarms during the day and to assist with recipients who have a heavy care burden. It was considered important that this be an educated and experienced person who could support the home care units in all types of tasks. As a first test, this person was set to serve two home care units and use a third unit as a control group *The intervention was implemented as planned*
Case 2	***Reflective work unit meetings with a facilitator*** The intervention work group prioritized the issue of a deteriorating social climate in the organization and decided upon an intervention to improve this situation. The group suggested that regular meetings be scheduled in each home care unit, where various aspects related to the work situation could be discussed and reflected upon. The aim was to get everyone within one unit to be more engaged in one’s own work situation as well as the work situation of one’s closest colleagues, and build a sense of community. The meetings would provide time to reflect together around the work content, the work environment and similar aspects. Two persons in each unit would be educated and serve as facilitators for the meeting, scheduled once a month *The intervention was not implemented as planned, due to a reorganization of both management and groups that started just after the intervention planning phase ended*
Case 3	***A new way to estimate travel time*** The work group focused on issues related to time slots dedicated to planning or travelling that often disappear due to other tasks. The travel time was currently based on an estimate from Google Maps that did not always match reality and did not take the type of building, or area, into account. If one needed to spend time on parking or climb three floors of stairs, this was not considered in the estimated travel time. The group suggested a dedicated time slot that each person could manage as best suited during the day, instead of letting the system automatically calculate and assign travel time. This time slot should cover travel time but could also be used for managing other tasks, including a phone call to a relative or to the medical nurse. The final intervention included an addition of two time slots of 15 min each that could be distributed as needed by the staff *The intervention was implemented as planned*
Case 4	***Raise work environment–related issues on the regular meeting agenda*** The main problem that was identified concerned time management, with a shortage of time between visits to different care recipients, in particular when they should visit a doctor, come home after a hospital stay or other activities that required more time than usual. Further, several employees perceived that there was an uneven distribution between the employees regarding care recipients who were physically and mentally demanding. Better time margins were requested. There was also a request that the entire staff group should agree on routines to help each other with physically and mentally demanding care recipients and support from the manager was requested to get this working. The intervention work group also discussed aids related to their physical work environment. The group did know that there were aids for care recipients. However, they appeared to be unaware of their own rights to have access to, for instance, lifting aids. As a consequence, they had not asked for them, despite the physical issues being brought up in the group. These discussions resulted in an identified need of a regular forum dedicated to discussing these very fundamental but important questions relating to work environment. The determined intervention was set to focus on introducing regular meetings where such issues could be handled *The intervention was partly implemented, but impossible to follow up due to a reorganization that dissolved the home care unit before the intervention could be evaluated*

### Intervention implementation phase: disturbances

As can be seen in Table 2, the interventions were implemented as planned in two out of four cases (case 1 and case 3). Based on the open-ended questions in the post-test questionnaire, we concluded that both of those interventions were perceived as successful by the personnel. The intervention in case 1 received an overall positive outcome from those answering the post-test questionnaire, while the one in case 3 received mainly positive and some neutral reactions. However, in the other two cases the implementation was derailed for various reasons. Several organizational changes, on several organizational levels, took place within the scope of the project, which acted as a main inhibitor for both implementing and evaluating the interventions.

#### Disturbances to the process

The Covid-19 pandemic has of course been a major disturbance in home care organizations since early 2020. For this study the pandemic was however not a major obstacle since the intervention planning phases in all cases ended during late 2018 or early 2019, with plenty of time to perform interventions before the start of the pandemic. Instead, reorganizations and changing of managers were the major factors influencing the process. During the four-year lifespan of the whole process (start-up and execution of the three phases of the framework), 14 managers were changed in the case organizations, and in the reference group consisting of external representatives there were 12 changes as well. Since home care managers were part of the reference group, some changes affected both the reference group and the case organizations. Besides changes in management, several bigger and smaller reorganizations took place. Cases 1, 2 and 4 went through reorganizations while the study was ongoing. In case 4 the organization was changed three times, with one bigger reorganization in the pre-intervention phase, including changes in management, and one local reorganization in the beginning of the intervention planning phase. The former resulted in case 4 being without a representative in the reference group over several meetings, and the latter in the intervention work group having to be restarted with a new group, since the members of the first version of the group were dissolved after the first meeting. This was the only disruption to the intervention planning phase in all fours cases. The planning phase ran smoothly in the other three organisations and also in case 4 once the new intervention work group was settled. The third reorganization in case 4 took place during the intervention implementation phase. As a consequence, the test group was dissolved before the intervention was followed up by the research group. Case 2 was also significantly affected by organizational turmoil. Most significantly, the first-line manager who had taken part in the intervention work group resigned at the end of the intervention planning phase, leaving the unit without management for three months. This was problematic, as it coincided with implementation of the intervention. When a new manager was finally in place in case 2, a reorganization took place. A consequence of this was that the commitment from management to the project and the planned intervention had to be reaffirmed. The pre-test questionnaire was distributed by the researchers, but the organization did not manage to start with the intervention during the scope of the project, although some initiatives were taken during autumn of 2020.

#### Systematic evaluation

Pre- and post-test questionnaires were performed in all four cases. All pre-test questionnaires were performed in 2019, after the intervention planning phase was finalized. Not all organizations managed to perform their intervention according to plan, but the post-test questionnaire was nevertheless distributed among the four cases. The high staff turnover, the high rate of other changes in the organizations, and a low response rate in the organizations that did not implement their intervention as planned, made it impossible to use the post-test questionnaire as a basis for a statistical evaluation of the specific interventions. It did, however, serve as a snapshot of the work environment in the organizations where the response rate was high enough, giving an implicit indication of the effects of all changes that had happened during the time since the first questionnaire. However, no within-subject analysis could be performed. The free-text questions in the post-test questionnaire from case 1 and case 3 did, on the other hand, provide rich data about the workers’ experiences of the specific intervention that could be used to draw some conclusions about the success of the intervention.

## Discussion

Research about interventions for improving the work environment in home care is sparse [15]. It is well known that this setting is problem-ridden, and in the light of the Covid-19 pandemic, this has become even more obvious. Adverse effects on healthcare workers have been discussed from a number of perspectives, including work organization and routines, staff continuity and protective equipment [45–48].

In this article, we present an action-oriented framework for participation-driven improvement of the work environment in home care that was implemented in four organizations. The implementation of the framework indicates that some parts of the framework work well; the pre-intervention phase served its purpose and elucidated the issues from science, practice and the labour market parties, showing that all information pointed in the same direction. In the implementation planning phase, the intervention work group process in each of the participating organizations also worked well. The work in the intervention work groups engaged the participants, and all four groups managed to identify and select one contextually relevant intervention to pursue. Other aspects of implementing the framework were not as straightforward, which is covered in the following discussion.

### Interventions in unstable organizations

Performing interventions in unstable environments is challenging [49, 50]. To introduce a new role (such as the resource person in case 1) or a new way of working (such as the procedure for handling travel time in case 3), several levels in the organization need to be involved. If managers on different levels are reorganized or replaced, the authorization of the intervention in the organization must be restarted. Furthermore, there is a risk that the proposed intervention will interfere with other changes that the new management wants to make (which was the situation in case 2).

The high turnover of people at all levels in the organization and the rush at which reorganizations were being implemented were surprising and had an important impact on the process. This happened even though the framework had been designed to meet some of the known challenges of doing participatory interventions in this context. As an example, the commitment and support from management was ensured *before* the project started, and *maintained* through the reference group and the first-line managers’ participation in the intervention work groups. Furthermore, the research project actually *compensated* the participating case organizations for time lost due to their involvement. Noticeably, the planned efforts to counter the effects of changes occurring outside the boundaries of the framework were not sufficient. And all changes that occurred outside the framework had an inhibiting effect on both the ability to implement interventions, and the ability to see differences between the pre- and post-test questionnaires related to the interventions. What we would like to emphasize is the importance of continuously evaluating the implementation process and keep it on track when potential obstacles occur. In the implementation of the framework in our study we offered an economic reimbursement for the organisations to manage to set of time for the activities in the study. Activities to follow the implementation process are suggested in our framework (see Fig. 2), but a plan for how to do this should preferably be decided upon before leaving the intervention work group phase. One way could be to let the intervention work group continue to have regular meetings during the implementation phase. Other methods could be to create some sort of focus groups that surveys the implementation. Having such groups could be a way to make sure that the engagement for the intervention persists, and a derailing of the process because of any organisational changes or other complications that might occur along the way, is hindered. In addition, being exposed to many concurrent changes may lead to increased stress and a deteriorating work situation [51], even if changes typically are introduced with good intentions and with the ambition to improve the work situation. Having a work group to guide the implementation of the intervention might help reduce such negative effects.

### Role of the manager

The role and attitude of the manager are important for the success of an implementation [38, 52, 53]. Whether a manager had been present at the intervention work group meetings or not was not a clear indicator for how the implementation went. However, manager continuity between the intervention planning phase and the intervention implementation phase was crucial. When no manager was present when the implementation phase started (as in case 2), the staff had no mandate to proceed with the intervention themselves. The high rate of change with regard to both management and the organization in case 4 can also be assumed to have affected the organization’s ability to commit to the project.

Having the manager as an active part in the intervention work group may be both beneficial and inhibiting. It is recommended to make sure that the intervention process is prioritized and that the chosen intervention can be implemented [49]. This is something management can contribute to. They can also provide an overview of issues that have been raised in the organization previously and solutions that have already been tested, or of issues that are currently being handled. However, the presence of a manager might also inhibit the other participants from speaking freely. Exactly how this was managed in the four cases varied somewhat, with the manager being part of all or only a few of the intervention work group meetings. In the design of the framework we determined, however, that the presence of a manager in the intervention work group would increase the likelihood of the suggested changes being mandated and the transition into the intervention implementation phase being smooth. This stance is supported by the failure to implement the intervention in case 2 when the manager quit just before the intervention implementation phase.

All involved managers where part of the reference group as well as the intervention work groups and, in the end, responsible for the implementation of the interventions. It was however, not the managers that took a leading role in the process of implementing the framework in their organizations. To make sure they support the participatory way of developing the organization, which is the fundamental idea of the framework, it could potentially have been useful with a guiding material that they could use to support them in their role at different stages of the process. The ideal scenario is that they take responsibility for the intervention process and work for more long-term organizational changes, which would be the ultimate goal of this type of process.

### Employee participation

Involving employees in change processes is important to ensure commitment to change and learning [23, 54]. A few studies of participative, action-oriented frameworks applied in the healthcare sector exist [55, 56]. However, they primarily address types of care and care personnel associated with a higher status than home care, that is, groups in which people traditionally have a voice and access to platforms that allow them to express their concerns (for instance, specialized care, and care involving physicians and registered nurses). The framework presented here, on the other hand, is designed specifically with the low status of the participants in mind, to truly empower them and to avoid *participation in name only*. That is, the aim is to ensure that the participants do not take part without gaining any influence, or that powerful participants completely dominate, with the result that the majority of participants are not being empowered [57]. Furthermore, the constellation of overlapping research group, reference group and intervention work group provides a possibility to help knowledge reach beyond the local context.

The degree and characteristics of involvement vary in participatory intervention processes [16, 19]. In the cases presented here the people in each intervention work group were very engaged in planning and designing the intervention. However, involving only a limited group of participants seldom leads to the desired organizational learning [54]. Since involvement and deployment of ideas do not merely happen, activities are necessary to facilitate participation beyond the most engaged participants [58]. Tasks were created to provide a structure for involving more members of the staff and hence to facilitate the group to involve more people in the activities. Nevertheless, it was not specified how the task should be executed, and it was not clear to what extent other people in the staff were involved in the end. Creating an explicit plan for how to perform the task could have helped, but is not a guarantee. Letting the facilitators, together with the participants in the group, execute the tasks in other forums where staff meet with each other could be another way forward.

### Work environment issues in theory and in practice

Home care work is associated with an increased risk for injuries, musculoskeletal disorders, high levels of sick leave and staff turnover, as well as perceived stress and high workload [3, 4, 6, 7]. When it comes to the work environment issues that were raised by the intervention work groups in this study, the focus was on planning, work organization, stress and social climate. Physical ergonomics and issues related to musculoskeletal strain were not brought up to any larger extent. One exception was in case 4, where this topic was raised and where group members seemed unaware of their own rights. This is possibly an effect of the high rate of change in the home care organizations, where staff members do not work long enough in a *stable* organization to develop an awareness of basic work environment rules. It is worth emphasizing that home care work is still heavy work causing a lot of sick leave due to physical strain [59, 60]. It is worrying that these issues were not identified as an area for improvement. This shows that despite something being well-established knowledge in scientific literature, it is not something that necessarily is a recognized problem in practice. Alternatively, it is considered part of the work and not something that can be improved. This could also be an indication of how the low status of the work affects the work environment in home care. In a sector associated with higher status, it would probably be harder to get away with the constant organizational changes that appear to characterize home care, since the employees would complain in such setting. Furthermore, the high staff turnover indicates that neither personnel nor managers are valued highly enough by the organizations to motivate them to invest sufficiently in retaining them.

### Systematic evaluation

Systematic evaluations of work environment interventions in home care are rare, and are difficult to conduct in complex real world organizations [36, 61]. As a consequence, opportunities for learning, both within and between organizations, are missing. The interventions developed in this study were evaluated with a pre-/post-test questionnaire, including intervention-specific as well as standardized work environment questions [41–43]. The conclusion is that addressing the effects of a specific intervention in home care by using standardized questionnaires, including general work environment–related aspects, is problematic. No within-subject variance can be detected due to lack of staff continuity, and any effects that can be seen in a generic pre-/post-test questionnaire cannot be related to the specific intervention due to the large number of organizational changes happening in parallel. Qualitative information gathered through open-ended questions was more useful in the two cases where the intervention had been implemented. Hence, in line with others, it is suggested to ask for subjective, in-depth descriptions of the staff’s experiences from the intervention, in combination with any additional relevant methods for measuring effects [61, 62]. Such information could be found using, for example, focus groups as a method for evaluation. A focus group comprising the intervention work group participants would seem like a natural part of the framework and could ideally be used. In the cases in the presented study, it was not possible to keep this long-term commitment due to staff turnover and reorganizations.

### Limitations

This study both presents a framework for participatory interventions in home care, founded in action research and learning, and tests it in four home care organizations. The complexity of this framework, including a group of researchers, a reference group and four home care organizations, as well as the cyclic process of reflection and action, tested in an unstable organizational context characterized by a high rate of change, makes it challenging to evaluate [63]. Exactly which aspects are central for success, and may have had an influence of the process, cannot easily be isolated. The use of multiple cases makes the identified concepts raised in the results more robust. Concurrent and retrospective conversations among the researchers (and likewise the intervention work group facilitators) reduced differences in individual interpretations.

Four specific cases were part of the study. These were all significant settings for the study, and they were selected with variations in certain dimensions but with similarities in the sense that they are all home care organizations in the south of Sweden and situated in a Swedish home care organizational context. The characteristics of this context are, however, similar to others regarding, for example, work environment issues and types of changes ongoing in the organization, and the study is therefore relevant in a wider context.

## Conclusions

This study contributes to the current knowledge about participatory interventions to improve the work environment in home care. This is a field of research that lags behind, although the need for improvement is critical. We present a framework for interventions identified and designed by the home care workers themselves, in facilitated intervention work groups, as a method to engage with and improve the work environment in their own organizations. The framework has an additional level, in the form of a reference group and a research group that work beyond the local context, both to spread knowledge about the developed interventions to a higher level and to provide insight into system-wide issues related to the work environment to the local organizations that are developing and implementing interventions. The framework was tested in four home care organizations with different levels of success. Parts of the framework were successful; the pre-intervention phase served its purpose and elucidated the issues from science, practice and the labour market parties, showing that all information pointed in the same direction; the intervention work group process in each one of the participating organizations also worked well.

Although Covid-19 has been dominating reports from home care lately, this was not the major obstacle in the study presented here. Instead, the huge number of changes occurring in the organizations and the quick pace at which they are implemented are identified as inhibiting factors for implementing the framework. With new teams, new management and new work routines constantly being introduced, the ideal conditions for implementing and evaluating a specific intervention are unfortunately not present. While theory regarding participative methods concordantly emphasizes organizational commitment and stability, it appears to be a reality that does not exist for home care. Since the work environment in home care is associated with many problems, the question is what we, as researchers, are going to do about it. In relation to the framework presented above, we have proposed some strategies that could increase the success rate by, for example, letting the work group continue to meet also during the intervention implementation phase in order to identify potential obstacles for success. However, the main issue for intervention-related research identified here, the instability of the organizational context, is hard to tackle on the level of the individual organization or home care unit. This is an issue that has to be approached on a much higher level.

## Data Availability

The datasets generated and/or analysed during the current study are not publicly available due to them containing information that could compromise research participants’ and organizations’ privacy/consent but are available from the corresponding author JP on reasonable request.

## Footnotes

1 A related professional group are the medical nurses employed by municipalities, who provide healthcare in the home. They are not the main focus of this study.
